# Modulation of Gut Microbial Biomarkers and Metabolites in Cancer Management by Tea Compounds

**DOI:** 10.3390/ijms25126348

**Published:** 2024-06-08

**Authors:** Hoi Kit Matthew Leung, Emily Kwun Kwan Lo, Fangfei Zhang, Felicianna, Marsena Jasiel Ismaiah, Congjia Chen, Hani El-Nezami

**Affiliations:** 1 School of Biological Sciences, University of Hong Kong, Pokfulam, Hong Kong SAR 999077, China; lhkm1997@connect.hku.hk (H.K.M.L.); emilylokwan@gmail.com (E.K.K.L.); u3567792@connect.hku.hk (F.Z.); feli19@connect.hku.hk (F.); marsena@hku.hk (M.J.I.); nedchen2@gmail.com (C.C.); 2 Institute of Public Health and Clinical Nutrition, School of Medicine, University of Eastern Finland, FI-70211 Kuopio, Finland

**Keywords:** tea compounds, cancer, gut microbiota, metabolites, microbiome targeted therapy

## Abstract

Cancers are causing millions of deaths and leaving a huge clinical and economic burden. High costs of cancer drugs are limiting their access to the growing number of cancer cases. The development of more affordable alternative therapy could reach more patients. As gut microbiota plays a significant role in the development and treatment of cancer, microbiome-targeted therapy has gained more attention in recent years. Dietary and natural compounds can modulate gut microbiota composition while providing broader and more accessible access to medicine. Tea compounds have been shown to have anti-cancer properties as well as modulate the gut microbiota and their related metabolites. However, there is no comprehensive review that focuses on the gut modulatory effects of tea compounds and their impact on reshaping the metabolic profiles, particularly in cancer models. In this review, the effects of different tea compounds on gut microbiota in cancer settings are discussed. Furthermore, the relationship between these modulated bacteria and their related metabolites, along with the mechanisms of how these changes led to cancer intervention are summarized.

## 1. Introduction

According to the WHO, there were an estimated 20 million new cancer cases and 9.7 million cancer-leading deaths in 2022 worldwide [1]. Among the cancer-induced deaths, lung cancer (18.7%) was the most death-leading cancer, followed by colorectal cancer (CRC) (9.3%) and liver cancer (7.8%). Given that cancer remains a major public health concern, advancement in early detection screening, diagnostic technology, and effective therapeutic options are therefore of urgent need [2]. At the same time, an enormous economic burden is posed to healthcare providers and patients in meeting clinical and daily needs [3]. With the high cost of cancer drug development and in particular of clinical trials, cancer drugs are unaffordable to many [4]. Therefore, the establishment of an alternative therapy that is effective and affordable would allow for more cancer patients to be treated.

Throughout the development of cancer, the commensal microbiota is disrupted, and the microbiome is altered into a configuration called gut dysbiosis [5,6]. An abundance of pathogens and tumor-promoting bacteria arise in cancers. *Helicobacter pylori*, *Fusobacterium nucleatum*, *Escherichia coli*, *Bacteroides fragilis*, and *Salmonella enterica* are several biomarkers found in the gut of cancerous individuals [6]. The rise of these pathogens promotes cancer through the production of pro-inflammatory cascades, enhanced cell proliferation, disrupted immunity, increased intestinal permeability, and elevated tissue damage from the increased reactive oxygen species. Meanwhile, the gut microbiota is responsible for the production of cancer-promoting and anti-cancer metabolites including fermentation products, protein catabolism, bile acids, and phytochemicals [7,8]. Short-chain fatty acids (SCFAs), and phenolic acids are beneficial metabolites found to be aiding in the battle against cancer while ammonia, polyamines, hydrogen sulfide (H_2_S), and secondary bile acids (BAs) are harmful metabolites that worsen the progression of cancers. Therefore, the modulation of the gut microbiota is suggested to be a therapeutic target given the strong influence of gut microbiota on cancer patients. Microbiome-targeted therapy can be carried out through fecal microbiota transplant, administration of probiotics and prebiotics, alteration of diet and lifestyle, or by administration of tailored antibiotics [9,10]. Nevertheless, the intense labor cost and efforts required for the tailored modulation of gut microbiota and fecal microbiota transplant limits the scalability of the technology. The induction of dietary compounds to target the gut microbiota is a more feasible option as it has broader and easier access at a relatively low cost.

In search of therapeutic options in cancer management, tea compounds are often studied by researchers for their bioactive nature [11,12,13]. Tea compounds are found to be bioactive with a diversity of polyphenols, polysaccharides, polypeptides, pigments, and alkaloids. The major compounds found in tea include epigallocatechin gallate (EGCG), caffeic acid (CaA), theabrownin (TB), quercetin (QUE), kaempferol, and spermidine (Spd). Alongside their antioxidant, anti-inflammatory and immune-modulatory effects, tea compounds have demonstrated proliferation, invasion, migration, metastasis, and angiogenesis inhibition and apoptosis promotion in cancer cells [12]. The anti-cancer effects were observed from gastrointestinal cancers like CRC, hepatocellular carcinoma (HCC), and gastric cancer (GC) to non-gastrointestinal cancers like breast cancer, prostate cancer, lung cancer, and cervical cancer [14,15,16,17,18,19,20]. Recent studies have revealed the relationship between tea compounds and gut microbiota [21]. Tea compounds were reported to induce prebiotic-like effects and alleviate gut dysbiosis, which regulates microbial diversity [21,22]. At the same time, gut microbiota are responsible for biotransforming and metabolizing tea compounds like tea catechins and tea theaflavins to perform their desired bioactivities [23].

Although the relationship between tea compounds and gut microbiota is being studied, the changes in gut microbiota in cancer settings induced by the different tea compounds are not all-inclusive. In addition, the modulation of gut microbiota-produced metabolites is of reciprocal importance in cancer management. Yet, the related information is scattered and limited. In this review, we recapitulate a more comprehensive summary of the gut microbiota modulatory effects of tea compounds and their induced changes on metabolite metabolism in cancer settings, aiming to emphasize the significance of tea compounds in the implementation of microbiome-targeted therapy of cancers.

## 2. Methods

A search of peer-reviewed articles on PudMed, Web of Science, and Google Scholar dated before March 2024 was performed. To identify research studies revealing the gut modulatory effects of tea compounds in cancer models, search terms used included “tea compounds”, “tea extracts”, “tea natural compound”, “cancer”, “tumor”, ”gut microbiota”, “microbiome”, “metabolites”, and “sequencing”. Search results were manually reviewed and only papers mentioning individual compounds with gut microbial or metabolomic sequencing methods performed in cancer models were included and further reviewed.

## 3. Gut Microbiota Modulatory Effects of Tea Compounds on Cancers

**Figure 1:**
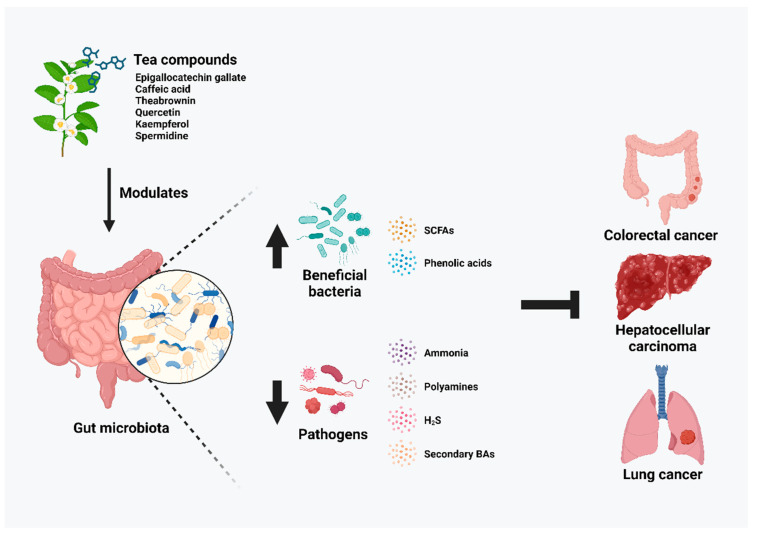
Summary of the gut microbial modulatory effect of tea compounds in colorectal cancer, hepatocellular carcinoma, and lung cancer.

During the initiation and development of cancer, the commensal community of microbiota is disrupted with a shift from symbiosis to dysbiosis, which directs the alterations of immunity and pathogenic responses in favor of tumorigenesis [24]. Therefore, the basics of microbiome-targeted therapy aim to reverse the dysbiosis conditions and restore healthy gut microbiota in order to alleviate the progression of cancer [25]. The significance of tea compound interventions in restoring the gut microbiota of cancer subjects is summarized in this review. The role of inflammation in cancer development and progression could not be further emphasized by being both the intrinsic and elicit factors of cancer [26]. Commensal and beneficial microbiota of the gut regulate such inflammatory responses by promoting anti-tumor immunity responses and achieving a repression of the heightened inflammatory status under cancerous conditions [27,28,29]. Bioactive compounds from tea have demonstrated gut microbiota modulatory effects in various cancers (Table 1) (Figure 1). 

### 3.1. Epigallocatechin Gallate (EGCG)

EGCG, (−)-epigallocatechin-3-gallate, is the most abundant catechin in green tea, accounting for around 60% of the total catechins [40]. EGCG is also considered the most active compound in green tea with anti-cancer, anti-proliferative, anti-cardiovascular, anti-obese, anti-diabetic, anti-allergic, antioxidant, neuroprotection, and gut health-promoting effects [40,41,42]. EGCG demonstrated CRC regulatory effects in an in vivo AOM/DSS study through the shift of gut microbiota composition. Wang et al. presented that EGCG reduced the number of precancerous lesions and tumors in AOM/DSS-induced CRC mice [30]. While no further biochemical analysis was carried out in the study, the possible cancer-promoting and cancer-alleviating effects were revealed. By 16s rRNA sequencing, it was revealed that the relative abundance of Bacteroides, Anaerotruncus, and Faecalibacterium were lowered while Clostridiaceae, Fusobacterium, Ruminococcus, Ochrobactrum, Veillonella, Desulfococcus, Enterobacteriaceae, Sulfurimonas, and Lactobacillus were increased by EGCG, which reversed the trend of the diseased model. For instance, Bacteroides is regarded as pathogenic with its ability to produce Bacteroides Fragilis toxin. The toxin was recognized to be cancer-promoting in humans, especially CRC through triggering COX-2-, IL-17-, and IL-6-related inflammation and activating STAT-3 and APC mutation [43,44,45]. Despite most of the studies studying Bacteroides, focused on the detrimental effects of pathogenic species Bacteroides fragilis [46,47,48], the genus was found to be more prevalent in obstructed CRC patients and CRC patients in general [49,50]. Meanwhile, Lactobacillus is a probiotic that inhibits mouse sarcoma, CRC, and bladder cancer. Whereas Lactobacillus is a well-known probiotic species, its strains exert anti-CRC and supplementary effects to the chemotherapeutics by enhancing treatment efficacy and reducing side effects [51,52,53]. The limitation of potential pathogenic bacteria and the enhancement of probiotics stabilized the gut microbiota composition, which accounted for the preventive effects of EGCG in CRC.

In a urethane-administered and high-fat diet (HFD)-exacerbated 16s cancer study, EGCG was found to ameliorate lung carcinogenesis and obesity with smaller tumors and lower weight gain [31]. It was reported that EGCG exerted anti-tumor effects through the reduction in leptin, STAT1, SCL7A11, and pro-inflammatory cytokine levels and the enhancement of anti-inflammatory cytokine levels. STAT1 is identified to activate SCL7A11, which suppresses ferroptosis [54]. The anti-tumor effect of EGCG in lung cancer was thus suggested to be triggering ferroptosis through the STAT1/SCL7A11 pathway. In addition, the obesity condition induced by HFD decreased Clostridia and increased Deltaproteobacteria and Epsilonproteobacteria, which contributed to the aggravation of lung tumorigenesis. Deltaproteobacteria was reported to be a producer of genotoxic H_2_S and associated with CRC when presented in the gut [55,56]. While for Epsilonproteobacteria, it was better known for its pathogenic members Campylobacter jejuni and Helicobacter pylori, which played significant roles in the progression of gastrointestinal cancer [57,58,59,60,61]. On the contrary, Clostridia was regarded as a promising treatment option for cancers including Clostridium-Directed Enzyme Prodrug Therapy (CDEPT), Clostridium-Directed Antibody Therapy (CDAT), and Combined Bacteriolytic Therapy (COBALT) [62,63,64,65]. EGCG reversed the gut microbiota alteration induced by HFD and along with regulation of STAT1-SLC7A11 ferroptosis, it alleviated the obesity-exacerbated lung cancer progression.

### 3.2. Caffeic Acid (CaA)

CaA, 3,4-dihydroxy-cinnamic acid, is a major hydroxycinnamic acid with effective antioxidant properties being found in tea, plants, and herbs [66,67]. CaA exerts multiple beneficial effects, such as anti-cancer, anti-proliferation, anti-inflammation, neuroprotection, anti-anxiolytic, and anti-obesity effects [67,68,69,70]. The effects of CaA on HCC were studied using the diethylnitrosamine (DEN) model by Zhang et al. [32]. CaA reduced HCC-related metabolic signatures and liver injury markers including alanine transaminase (ALT), aspartate aminotransferase (AST), alkaline phosphatase (ALP), total BAs, total cholesterol, high-density lipoprotein cholesterol, and low-density lipoprotein cholesterol in DEN-treated rats. Such changes were suggested to be contributed by the modulatory effects on gut microbiota and metabolites. CaA alleviated HCC by returning altered cancer-related metabolites to normal levels, which were correlated to the modulation of gut microbiota. CaA reversed the increased *Rumincoccaceae UCG-004* and decreased *Lachnospiraceae incertae sedis*, and *Prevotella 9* in HCC rats. The uprise in *Ruminococcus* and the reduction in *Lachnospiraceae* are suggested to be gut microbiota signatures of non-alcoholic fatty liver disease (NAFLD) and non-alcoholic steatohepatitis (NASH). In addition, the metabolic profiles were modified by CaA, and 41 metabolites were returned to normal level. Among these metabolites, bilirubin, L-tyrosine, L-methionine, and ethanolamine were correlated to the above three bacteria, suggesting their potential to be developed into therapeutic targets. Bilirubin and tyrosine levels were associated with the AFP, AST, ALT, and oxidative damage levels in HCC subjects, while L-methionine is detrimental to hepatocyte functions and ethanolamine is a universal marker of cancer. Nevertheless, *Lachnospiraceae* was suggested to be capable of metabolizing tyrosine, and *Prevotella* could remove L-methionine. CaA elevated *Lachnospiraceae* and *Prevotella*, which could metabolize the detrimental L-tyrosine and L-methionine, respectively [71,72]. However, there is a lack of other studies that discuss the topic. Overall, through gut microbiota modulation, CaA returned cancer-related metabolites to normal levels and achieved HCC-inhibitory effects.

### 3.3. Theabrownin (TB)

TB is a microbial fermented polyphenol found in dark tea like Pu-erh tea and Fu Brick tea [73,74]. The health benefits of TB include preventing tumors, improving immunity, anti-inflammation, alleviating obesity and metabolic syndromes, and ability to improve gut microbiota [73,74,75,76,77,78]. TB demonstrated anti-tumorigenesis effects on CRC in the AOM/DSS model [33]. The administration of TB reduced the tumor count and spleen weight of AOM/DSS mice while suppressing tumor cell proliferation and promoting apoptosis through the suppression of the phosphoinositide 3 kinase (PI3K)/protein kinase B (Akt)/mammalian target of rapamycin (mTOR) pathway. TB reduced the relative abundance of *Bacteroidceae* family and *Bacteroides* genus and elevated the *Prevotellaceae* family, *Alloprevotella* genus, and *Romboutsia* genus. In addition, the study also revealed that *Anaeroplasma* and *Rikenellaceae_RC9_gut_group* genera were negatively correlated to the total tumor count while the downregulated *Bacteroides* genus was positively correlated to the total tumor count. The *Bacteroidceae* family and *Bacteroides* genus were reported to be CRC-related and worsen tumor multiplicity and burden in AOM/DSS mice. Meanwhile, the *Romboutsia* and *Anaeroplasma* genera are reported to have immunity-strengthening and anti-inflammatory effects. *Romboutsia* demonstrated an inverse correlation with cellular inflammatory factors and pro-inflammatory TNF-α, IL-6, and IL-17 cytokines but not for anti-inflammatory IL-10 in colitis and rheumatoid arthritis models [79,80]. *Anaeroplasma* was reported to correlate to downregulation of the pro-inflammatory IL1a, IL6, IL12a, IL12b, and IL17a and the upregulation of the anti-inflammatory IL10 cytokines in CRC mice [81]. Similarly, *Anaeroplasma* was reduced in CRC mice but was restored after the treatment of resveratrol [82]. The reshaping of gut microbiota induced by TB favored an anti-inflammatory cytokine profile and immunity-strengthening effects to achieve CRC management.

### 3.4. Quercetin (QUE)

QUE is a flavonoid found in black tea, fruits, and vegetables with anti-cancer, anti-inflammatory, anti-diabetic, cardiovascular-protecting, and gut microbiota modulatory effects [83,84,85,86]. The gut microbiota modulatory effects of quercetin in CRC and HCC have extensively been discussed [34,35,36]. In an ApcMin/+ mice model, QUE along with microencapsulated probiotics *Bifidobacterium bifidum* and *Lactobacillus gasseri* inhibited the development of CRC with reduced ACF and adenoma and intestinal bleeding by inhibiting the canonical Wnt/β-catenin signaling [34]. Wnt/β-catenin-related genes were found to be reduced to halves of the level of ApcMin/+ mice after the treatment. In addition, by agar plating diluted fecal samples, microbial counts of *Enterobacteriaceae* and coliform bacteria were significantly reduced while *Lactobacillus* and *Bifidobacterium* increased. *Enterobacteriaceae* was suggested to induce strong pro-inflammatory effects and the coliform was related to CRC progression. *Enterobacteriaceae* was reported to be a major gut dysbiosis contributor by enhancing inflammatory responses in the gut, in which the expansion of *Enterobacteriaceae* was often reported in IBD, obesity, colitis, and CRC [87,88,89]. *Lactobacillus* and *Bifidobacterium* are known for promoting intestinal functions and combating the development of CRC. The addition of QUE alongside the microencapsulated probiotics further enhanced the level of Lactobacillus and Bifidobacterium suggesting the important role of QUE in boosting the growth of these beneficial bacteria in the regulation of Wnt/β-catenin signaling.

While the anti-cancer effects of QUE on CRC were reported and its gut microbiota modulation in CRC was discussed, Catalán et al. studied the effect of QUE’s microbial metabolite 3,4-dihydroxyphenylacetic acid (3,4HPAA) on CRC [36]. In both cancer and normal colon epithelial cell lines, 3,4HPAA prohibited cell proliferation and favored caspase 3 activity and cytochrome c release. *Eubacterium ramulus* is responsible for the production of 3,4HPAA from quercetin as well as the production of butyrate when co-treated with *B. thetaiotaomicron* [90]. Interestingly, the metabolite established stronger effects than the parent QUE, illustrating the critical role of 3,4HPAA and microbial metabolism in QUE’s anti-CRC effects.

Apart from CRC management, QUE also presented HCC regulatory effects by reshaping gut microbiota. QUE combined with the use of anti-programmed cell death 1 (anti-PD-1) antibody reduced necrosis, fibrosis, and PD-L1 expression in the liver of an orthotopically transplanted HCC model [35]. The treatment achieved HCC management by improving the overall macrophage immunity, in which the expressions of CD8a, CD4, CD11b, IL-10, and IFN-γ were elevated, and expressions of IL-4, IL-6, and Toll-like receptor 4 (TLR4) declined. The combination therapy decreased the abundance of Bacteroidetes phyla and increased *Firmicutes*, *Actinobacteria*, and *Verrucomicrobiota* phyla and *Dubosiella* and *Akkermansia* genera. *Akkermansia* was recognized for improving immunity and preventing tumor growth in cancer models. The improvement in *Firmicutes/Bacteroidetes* ratio indicated the ecological balance of gut microbiota. A lower *Firmicutes/Bacteroidetes* ratio was often seen in cancer subjects and as an indication of dysbiosis within the gastrointestinal tract [91,92]. Taken together, these studies suggested the potential of QUE as a microbiome-targeted therapy when applied to existing therapeutic options.

### 3.5. Kaempferol

Similar to QUE, kaempferol is another natural-forming bioactive flavonoid found in tea, fruits, and vegetables that serves multiple health-promoting effects including anti-cancer, anti-inflammation, neuroprotection, anti-diabetic, anti-obese, hepatoprotection, and microbiota balancing effects [93,94,95,96,97,98]. The study of Li et al. specifically studied the relationship between the bile acid–gut microbiota axis and CRC with the administration of kaempferol [37]. Kaempferol significantly reduced tumor burden, improved damaged intestinal barrier, and downregulated Ki67 and leucine-rich repeat-containing G-protein-coupled receptor 5 (LGR5) expressions in ApcMin/+ mice. Regarding the BA metabolism, kaempferol elevated CDCA and 12α-hydroxylated BAs by upregulating the expression BA regulator, including CYP27A1, CYP8B1, and FXR, and the BA transporter, including BSEP and MRP2. The reversal of BA imbalance was suggested to be influencing the composition of gut microbiota. Kaempferol elevated the abundance of *Actinobacteria* and *Verrucomicrobia* phyla as well as several beneficial bacteria species including *Akkermansia muciniphila*, *Bacteroides acidifaciens*, *Barnesiella* spp., and *Bifidobacterium choerinum*, which improves the efficacy of anti-cancer drugs. In addition, *Parabacteroides goldsteinii*, which was related to alleviate obesity through enhancing BA metabolism, was also elevated. On the contrary, the abundance of pathogens, including *Anaerostipes*, *Desulfovibrio*, *Helicobacter*, *Lachnospira*, *Marvinbryantia*, *Oscillibacter*, *Roseburia*, and *Ruminococcus* were reduced. Furthermore, by studying gut microbiota predicted pathways through PICRUSt (1.1.4) analysis, kaempferol suppressed lipid metabolism, amino acid metabolism, and energy metabolism, illustrating the limiting effects on the energy expenditure in ApcMin/+ mice. Also, secondary BA synthesis pathways were downregulated as correlated to the decreased secondary BA metabolic bacteria including *Clostridium lavalense*, *Eubacterium desmolans*, *Eubacterium rectale*, *Intestinimonas butyriciporducens*, *Lachnoclostridium hylemonae*, and *Pseudoflavonifractor bacteroides capillosus*. The abundance of secondary BA metabolic bacteria *Clostridium lavalense*, *Eubacterium desmolans*, *Eubacterium rectale*, *Intestinimonas butyriciporducens*, *Lachnoclostridium hylemonae*, and *Pseudoflavonifractor bacteroides capillosus* were downregulated as well as the secondary BA synthesis pathways. *Eubacterium* spp. including *E. desmolans* and *E. rectale* contribute to BA synthesis and homeostasis including secondary BA production [99]. *Lachnoclostridium hylemonae* is capable of converting the primary CA and CDCA to the secondary DCA and LCA, respectively [100], while *Pseudoflavonifractor bacteroides capillosus* was suggested to be a major bacterium for the transformation of LCA [101]. The study emphasized the role of gut microbiota homeostasis and bile acid regulation in the regulation of CRC by kaempferol.

Kaempferol also exhibited inhibitory effects on lung cancers in xenograft mice models. Lewis lung carcinoma (LLC) cell-induced tumors shrunk with the administration of kaempferol with the activation of cytotoxic T cells, natural killer cells, and macrophages [38]. The anti-LLC effects of kaempferol-induced gut microbiota modulation were studied by performing 16s rRNA analysis. Kaempferol significantly elevated the abundances of the *Lachnospiraceae* and *Bacteroidaceae* family, and the *Lactobacillus* and *Bacteroides* genera. *Lactobacillus* contains species that facilitate anti-tumor activity in lung cancer and provide immunoregulatory functions. These bacteria were found to be positively correlated to the elevation of CD8+/INFγ+, CD68+/INFγ+, and CD49b+/CD107a+ cells under correlation analysis. Furthermore, from the metabolic pathways prediction in KEGG, kaempferol upregulated the carbohydrate metabolism, lipid metabolism, and nucleotide metabolism pathways and downregulated the signal transduction (environmental information processing), amino acid metabolism, and global and overview map (metabolism) pathways, which were suggested to activate immune cells. The *Lactobacillus* species is one of the biggest groups of probiotics, and has been well studied [102]. In terms of cancer studies, *Lactobacillus* exerted immunomodulatory effects including stimulating the immune system, regulating cytokine release, enhancing the phagocytic activity of macrophages and NK cell activity, secreting of immunoglobulin A, and inducing specific markers on the immune cell surface [103]. In terms of immunomodulation, *Bacteroides*-expressed capsular polysaccharide A (PSA) is well-documented [43]. PSA was reported to induce CD4+ and CD8+ T cell-dependent immune responses, regulate the Th1/Th2 balance, and activate anti-inflammatory IL-10 [104]. To sum up, kaempferol promotes immunoregulation by activating T cells, NK cells, and other immune cells through gut microbiota modulation and achieves anti-cancer effects in LLC.

### 3.6. Spermidine (Spd)

Spd is one of the major polyamines found in tea, wheat, nuts, soybeans, fruits, and vegetables. The health effects of Spd include life span extension, anti-cancer, anti-inflammation, neuroprotection, hypertension-related disease protection, and anti-obese effects through gut microbiota improvements [105,106,107,108,109,110]. Although polyamines are generally linked to cancer progression, Spd exerts dual effects in cancer progression, where a higher uptake of Spd is suggested to suppress tumor progression [107,111]. Gobert et al. presented the protective effects of Spd on CRC in both the AOM/DSS and Apc loss model [39]. It was revealed that the deletion of the spermine oxidase (Smox), which catalyzes the conversion from spermine (Spm) to Spd, worsens CRC in the AOM/DSS model. The administration of Spd prevented the carcinogenesis of AOM/DSS in both wildtype and Smox−/− by ameliorating the number of tumors, total tumor burden per colon, tumor size, and the number of histologic adenomas. The loss of Smox and AOM/DSS treatment was found to increase α-defensins, which was associated with colitis and CRC. Spd treatment significantly reduced the expression of genes encoding for α-defensins. Moreover, Spd reversed the reduced *Prevotella* and increased *Proteobacteria* and *Deferribacteres* in Smox−/− mice. Reduced *Prevotella* and increased *Proteobacteria* and *Deferribacteres* were suggested to be associated with colitis. *Proteobacteria* and *Deferribacteres* were both related to inflammatory-related diseases and demonstrated their pathogenicity in cardiovascular diseases, obesity, NASH, IBD, and colitis [112,113,114,115,116]. The reversal effect of Spd was therefore proposed to be important in preventing the development of colitis and CRC. In addition, Spd inhibited tumorigenesis in the Apc loss model with a reduced number of tumors. Therefore, it was suggested that Spd supplementation prevented colitis and CRC by hindering α-defensin expression and maintaining a protective effect through modulating gut microbiota composition.

## 4. Tea Compound-Modified Microbiota Regulates Cancer-Related Microbial-Derived Metabolites

**Figure 2:**
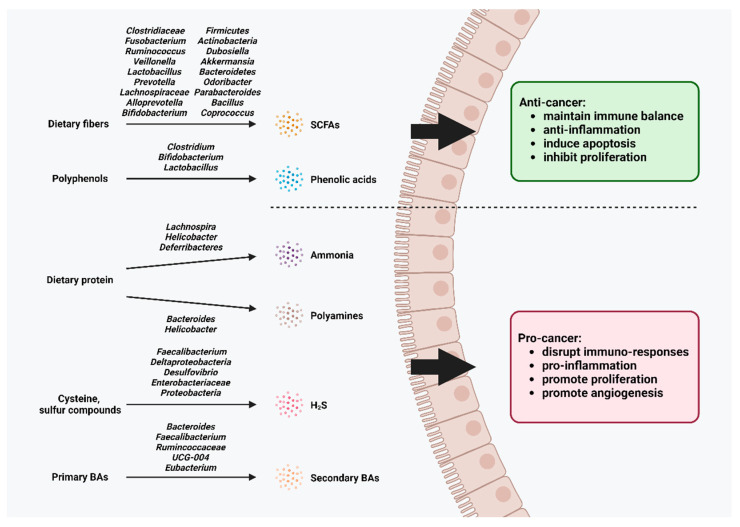
Summary of metabolites synthesized by tea compounds modulated gut microbiota in cancer management.

Apart from its direct impact on gut microbiome structure, gut microbiota also impacts the host’s health by producing microbiota-derived metabolites [117,118]. Among previously mentioned tea-altered gut microbes, they have previously been found to correlate with cancer-related metabolites including ammonia, polyamines, H_2_S, and secondary BAs. Furthermore, the administration of tea compounds was also shown to promote beneficial gut microbe-derived metabolites, SCFAs, and phenolic acids and downregulate the microbiota correlates to cancerous metabolite production (Figure 2).

### 4.1. Short-Chain Fatty Acids (SCFAs)

SCFAs are fermented products from the saccharolytic fermentation of non-digestible carbohydrates [119]. SCFAs are the major metabolites produced in the gut that have promising effects in the management of breast, colorectal, lung, liver, and prostate cancers [120,121,122]. In particular, acetate, propionate, and butyrate are the major SCFAs performing anti-cancer effects [123]. SCFAs regulate cancer pathogenesis by maintaining inflammatory cytokine balance, improving the intestinal mucosal barrier, boosting intestinal immunology, inhibiting epigenetic regulators on oncogenic cell signaling pathways, and blocking cancer stem cell proliferation [120,123]. Upon absorption, SCFAs enter cells through the G-protein-coupled receptors (GPCRs), specifically activating GPR109A and GPR41/43 and inhibiting histone deacetylases (HDACs) [123,124]. The activation of GPR109A was suggested to contribute to the differentiation of T reg cells and anti-inflammatory IL-10, while GPR41/43 contributed to activating dendritic cell, macrophage, and antibody production. In addition, the high dose of SCFAs inhibited HDAC, which stimulated apoptosis and limited the proliferation of cancer cells. Taken together, both acute and chronic inflammatory responses and tumor growth were suppressed by SCFAs, leading it to be an important metabolite for combating cancer.

All tea compounds included in this review have demonstrated gut microbiota modulations that are related to the production of SCFAs. *Clostridiaceae*, *Fusobacterium*, *Ruminococcus*, *Veillonella*, and *Lactobacillus* are SCFA-producing bacterial genera elevated by EGCG. The abundance of *Clostridiaceae* was found to be lowered in the early stages of CRC [125]. The butyrate-producing properties of *Clostridiaceae* suggested that it could provide probiotic functions to the homeostasis of the intestine [126]. *Fusobacterium* was reported as an SCFA-secreting species [127,128,129]. *Ruminococcus* is also well known as a butyrate producer and is associated with higher fecal SCFA concentration [130,131,132]. The bacteria and other butyrate producers were found to be reduced in HCC and gastrointestinal cancers [133]. *Veillonella*, a propionate producer, performed anti-cancer functions in oral squamous cell carcinoma [134,135,136]. The SCFA-producing properties of Lactobacillus are important contributors to its probiotic nature [137]. For instance, *Lactobacillus fermentum NCIMB-5221* and *8829* were the most potent in producing SCFAs from a screening of *Lactobacillus* strains [138].

*Prevotella* is elevated by CaA and Spd, while *Lachnospiraceae* is elevated by CaA as well. *Lachnospiraceae* strains were described to have SCFA production properties, and positive correlations between the bacteria and SCFAs were found in ulcerative colitis settings [139,140,141]. *Prevotella* is an SCFA-producing bacterium. *Prevotella* facilitates the breakdown and fermentation of dietary fiber and polysaccharides, resulting production of SCFAs, mostly propionate [123,142,143].

On the other hand, the TB-increased *Prevotellaceae* family and *Alloprevotella* genus are SCFA producers that regulate the occurrence of CRC. Previous studies reported a positive relationship between SCFA production and *Prevotellaceae* [129,144]. *Alloprevotella* is also a major butyrate producer that supports the health of the host and inhibits the growth of pathogens [145].

The SCFA-producing *Bifidobacterium*, *Firmicutes*, *Actinobacteria*, *Dubosiella*, *Lactobacillus*, and *Akkermansia* are increased by QUE treatment. *Bifidobacterium* is related to the production of acetate, propionate, and lactate [146]. *Firmicutes* are SCFA producers whose abundance is intensely reduced during tumor growth [147,148]. *Actinobacteria* also showed butyrate production properties [149]. *Dubosiella* was found to be positively correlated with SCFAs from a study on the prebiotic properties of *Oudemansiella raphanipes* mushroom polysaccharides [150]. *Akkermansia*, which belongs to the *Verrucomicrobiota* phylum, produces SCFAs that prevent colitis through anti-inflammatory responses [151].

Kaempferol increased the abundance of Bacteroidetes, Bifidobacterium, Odoribacter, Ruminococcus, Parabacteroides, Bacillus, Firmicutes, Alloprevotella, Actinobacteria, Akkermansia, Lachnospiraceae, Coprococcus, and Lactobacillus. Bacteroidetes, such as Bacteroides, could utilize dietary polysaccharides to produce SCFAs [152]. Odoribacter is part of the healthy and balanced gut microbiota with its capability to produce SCFAs [153]. Parabacteroides secretes SCFAs, acetate in particular, which helps to alleviate heparinase-exacerbated acute pancreatitis [154,155]. Bacillus spp. are SCFA producers for which Bacillus subtilis could improve butyrate secretion and support the growth of other butyrate-producing bacteria as well as improve the mucosal barrier [156,157]. Coprococcus spp. produces butyrate, and the uptake process of butyrate in colonocytes secretes glucagon-like peptide 1 (GLP-1) and anorexigenic peptide YY (PYY), which provide protective neurological effects at the same time [158].

### 4.2. Phenolic acids

The gut microbiota plays significant roles in the metabolism, transformation, and synthesis of phenolic acids [159,160]. The previously mentioned anti-cancer CaA and 3,4HPAA are both phenolics synthesized through the biotransformation of gut microbiota. While the actual mechanism of anti-cancer effects varies between compounds, phenolic acids have demonstrated anti-cancer properties by promoting apoptosis, antioxidant, tumor necrosis, anti-inflammatory, and estrogenic effects [161,162]. The administration of phenolic acids promotes the generation of reactive oxygen species (ROS) above the threshold level within cancer cells and triggers cancer cell death. At the same time, the oncogenic nuclear factor kappa-light-chain-enhancer of activated B cells (NF-κB), mitogen-activated protein kinases (MAPK), and PI3K/AKT signaling cascades were limited while tumor suppressor P53 (p53), cyclin-dependent kinase inhibitor 1 (p21), and cyclin-dependent kinase inhibitor 1B (p27) were elevated. Proliferative interleukin 6 (IL-6) and tumor necrosis factor alpha (TNF-a) cytokines were also found to be downregulated by phenolic acids.

EGCG elevated *Clostridium*; QUE and kaempferol elevated *Bifidobacterium*; and *Lactobacillus,* which was elevated by EGCG, QUE, and Kaempferol, are related to the production of beneficial phenolic acids. Apart from inhibiting intestinal tumors through the production of SCFAs [163], *Clostridium* also produces lactic acids, phenyllactic acids, 2-hydroxybuturic acids, and phenylpropionic and p-hydrophenylpropionic acids, which maintain health and possess antioxidant properties [164,165]. Not only do polyphenols elevate and support the growth of *Bifidobacterium*, but the bacteria can also metabolize phenolic acids [166,167,168]. *Lactobacillus* are important in the metabolism and degradation of caffeic acid, p-coumaric acid, ferulic acid, and gallic acid, which in the process facilitates energy regeneration with the creation of nicotinamide adenine dinucleotide (NAD+) [169].

### 4.3. Ammonia

Ammonia is another detrimental metabolite that is produced during gut dysbiosis. Dietary protein is degraded into toxic byproducts like ammonia, phenols, and cresols by the gut microbiota in the distal colon [170,171,172]. The accumulation of ammonia worsens the progression of cancer through the recycling of ammonia for growth and inhibiting the activation of T cells. Although ammonia is generally treated as a metabolic waste, cancer cells could recycle ammonia to maximize nitrogen utilization, which supports tumor biomass [173]. Instead of being expelled as urea, ammonia accumulates in the microtumor environment and is used to synthesize amino acids to support tumor growth. In addition, tumor ammonia accumulation induces T cell exhaustion and hinders the efficiency of immunotherapy [174]. The accumulation of ammonia leads to exhaustion, the death of T cells, and the upregulation of PD-1, which hinder the anti-tumor ability of one’s immunity system.

The production of ammonia is suggested to be prevented with the treatment of QUE, kaempferol and Spd. QUE and kaempferol have been found to increase *Bifidobacterium*, which has demonstrated ammonia assimilation properties. Oral administration of *B. bifidum YIT4069* prevented ammonia production and lowered cecal ammonia level [175]. Meanwhile, kaempferol lowered the abundance of *Lachnospira* and *Helicobacter*. Exposure to ammonia was reported to increase pathogens including *Lachnospira* [176]. *Helicobacter* is well known for its GC-causing member *Helicobacter pylori*. The ammonia produced by *H. pylori* was reported to worsen hepatic cirrhosis and damage human intestinal epithelial cells [177,178]. Spd decreased the abundance of *Deferribacteres*; its member *Mucispirillum schaedleri* blooms under inflammatory conditions [179]. Similar to other members of the *Deferribacteres* phylum, the bacteria have the ability to reduce the electron acceptor nitrate to ammonia given that nitrate is increased in inflamed gut.

### 4.4. Polyamines

Apart from dietary sources, microbial fermentation is another major source of polyamines in humans [180]. Polyamines are responsible for cell growth, proliferation synthesis of protein and nucleic acids, and regulating cell membrane stability, yet polyamine metabolism is often dysregulated in cancers [181,182]. The increased concentration of polyamines is related to tumorigenesis and cancer risk. Dysregulated polyamine metabolism could promote cancer through the activation of major oncogenic signaling pathways like MYC proto-oncogene, BHLH transcription factor (MYC), rat sarcoma virus/rapidly accelerated fibrosarcoma (RAS/RAF), p53, AKT/mTOR, Rac/Ras homolog family member A (Rac/RhoA), activator protein 1 (JUN/FOS) and Lin-28 homolog A (LIN28).

EGCG, TB, and kaempferol reduced polyamine-related *Bacteroides* and *Helicobacter*, respectively. *Bacteroides fragilis* was suggested to promote colon cancer with catabolism of polyamines [183]. Upon *H. pylori* infection, the polyamine level elevates in gastric tissues, which inhibits inducible nitric oxide synthase (iNOS)-mediated immunity [184].

### 4.5. Hydrogen Sulfide (H_2_S)

The primary sources of H_2_S in the human body include production from endogenous enzymes such as cystathionine γ-lyase (CSE), cystathionine β-synthase (CBS), and 3-mercaptopyruvate sulfurtransferase (3-MST), as well as byproducts of microbial metabolism from gut microbiota, including sulfate-reducing bacteria [185]. Taurine or glycine are often conjugated to primary BAs, and throughout the conversion of secondary BAs, taurine and glycine are cleaved as residues and reduced to H_2_S by sulfate-reducing bacteria [8]. At the same time, a diet with high meat consumption promotes taurine conjugation, which supports the bloom of H_2_S producing sulfate-reducing bacteria, and thus the capacity for gut microbiota to deconjugate taurine was suggested to be an indication of CRC risk [7,8]. Although H_2_S exerts dual properties on cancer, its anti-cancer effects are only observed when cancer cells are exposed to high concentrations of H_2_S. Most of the time, H_2_S is tumor-promoting through the enhancement of cellular bioenergetics; activation of oncogenic and invasive signaling pathways; and increased tumor angiogenesis [186]. H_2_S exerts anti-apoptosis properties in the tumor site by activating the NF-κB, MAPK, and PI3K/AKT signaling pathways [187]. At the same time, the activation of NF-κB promotes cancer metastasis, and the activation of the MAPK and PI3K/AKT pathways favors angiogenesis, which supports further tumor growth.

EGCG demonstrated inhibitory effects to H_2_S-related *Faecalibacterium* and *Deltaproteobacteria*. *Faecalibacterium* was found to be significantly elevated with the treatment of H_2_S [188]. Deltaproteobacteria is a sulfate-reducing bacterium that could produce H_2_S as a byproduct [189,190]. Throughout the respiration of *Deltaproteobacteria*, taurine is used to produce ammonia, acetate, and sulfide [191,192]. Kaempferol reduced *Desulfovibrio*, a sulfate-reducing bacterium that is known for its H_2_S-producing properties [193,194]. A high level of H_2_S was suggested to be toxic to the colonocytes. It was also reported that *H. pylori* promotes endogenous H_2_S production, which is involved in the buildup of gastric mucosal diseases [195]. Also, QUE diminished *Enterobacteriaceae*, which is related to H_2_S, taurine, and secondary BA production [196]. *Enterobacteriaceae*-mediated taurine was positively associated with the development of IBD [88,197]. In addition, Spd has been found to reduce the abundance of the *Proteobacteria* phylum. It is worth noting that many of the prominent H_2_S producers belong to this phylum [198]. On the other hand, QUE and kaempferol also increased *Akkermansia*, which consumes H_2_S to produce cysteine and limit the damaging effects posed to the host [199].

### 4.6. Secondary Bile Acids (BAs)

The major secondary BAs, lithocholic acid (LCA) and deoxycholic acid (DCA), were found to be cancer-promoting in cholangiocarcinoma, CRC, GC, HCC, hypopharyngeal squamous cell carcinoma, non-small cell lung cancer, esophageal adenocarcinoma, and pancreatic cancer [200,201,202,203,204]. Secondary BAs could promote cancer through the inhibition of apoptosis in cancer cells, activating oncogenic signaling pathways, inducing inflammation, and inhibiting the functions of immune cells. Secondary BAs are prone to elevated pro-inflammatory M1 phenotype cytokines like TNF-a and IL-6 over anti-inflammatory M2 phenotypes like IL-10 [201]. DCA inhibits apoptosis by upregulating anti-apoptotic BCL-XL and activating the NF-κB signaling pathway while LCA activates the proliferative ERK1/2 pathways [202].

Secondary BA-related *Bacteroides* were reduced by EGCG, TB, and Kaempferol, and *Faecalibacterium* was reduced by EGCG. The level of secondary BAs, especially DCA, was reported to be positively correlated with the abundance of *Bacteroides* [205]. The member *Faecalibacterium prausnitzii* was positively correlated to secondary BAs while being negatively correlated to primary BAs [206,207]. In addition, *Rumincoccaceae UCG-004* was reported to be associated with an increase in fecal cholic acid excretion [32,208]. The bacteria were reduced by CaA. Meanwhile, kaempferol reduced *Eubacterium*, one of the bacterial groups that convert primary BAs to secondary BAs, which was suggested to be related to CRC progression [209,210].

## 5. Limitations on Currently Available Studies

The reviewed articles exemplified how tea compounds contribute to cancer management through gut microbiota and microbial metabolite modulation. Nevertheless, these studies are limited to gastrointestinal CRC, HCC, and lung cancer, which is often the metastasis site for bowel cancers [211]. Recent studies have suggested that gut microbiota plays significant roles in non-gastrointestinal cancers as well in terms of cancer development, the influence on immunity, interaction with anti-cancer therapy, and the production of microbial metabolites [212,213,214]. Moving forward from in vitro studies, in vivo evidence on how tea compound-induced microbiota modulation impacts non-gastrointestinal cancers would deepen the understanding of its anti-cancer effects. In addition, many current studies were restricted to the use of 16S rRNA sequencing, which could not achieve maximum resolution at the genus level, as it could only identify a limited region of genes [215]. Shotgun metagenomic sequencing and next-generation sequencing could be applied to provide a more in-depth understanding of the modulation of gut microbiota at species and strain level and thus provide the clarity to assess the efficacy and safety of the given microbiota [216,217]. Whereas there are a limited number of studies that examine gut microbiota composition through sequencing, studies that also assess gut microbiota-derived metabolite profiles are scant. On one hand, gut microbiota-derived metabolites establish a connection between the modulation of gut microbiota and cancer initiation and progression. On the other hand, they also demonstrate the potential in the development of cancer treatment [212,218]. Further study is needed to further elucidate the full potential of tea bioactive compounds as a potential microbiome modulator for cancer therapy, especially with the confirmation studies on the anti-cancer effects of the microbial biomarkers identified from sequencing methods with higher-resolution power.

## 6. Implication of Findings in Clinical Practice

With the increased availability of information and confirmation of the causal relationship between cancer and the identified gut microbiota and metabolites in future studies, the implications of tea compounds in the microbiome-targeted therapy of cancers would be favored given their economic and easy-access nature. Such findings could be directly implemented as cancer therapeutic agents and cooperated with adjuvant therapy to enhance efficacy and reduce the toxicity of standard chemotherapy [212,219]. Apart from directly establishing their anti-cancer effects as mentioned, tea compounds are also important adjuvant agents in improving the efficacy of chemo drugs while reducing their side effects. For example, EGCG was suggested to induce chemo-sensitization while protecting against chemotherapy side effects including gastrointestinal disorders, disruption of immune systems, and nephrotoxicity [220]. At the same time, the significance of gut microbiota in preventing chemotherapy-induced toxicity was proven in a fecal microbiota transplant study [221].

## 7. Conclusions

**Figure 3:**
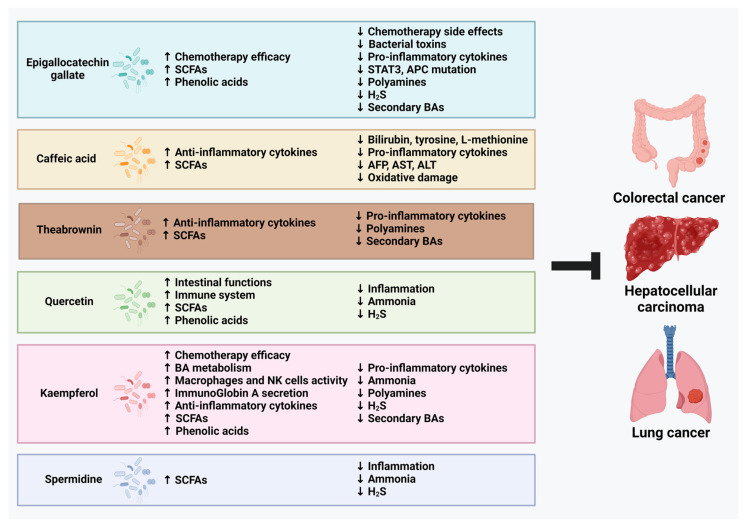
Summary of mechanisms involved in the modulation of gut microbiota by tea compounds in cancer management.

Tea compounds have been shown to reverse gut dysbiosis and improve the microbiota profile in in vivo studies. These compounds can suppress the heightened inflammation and dysregulation of oncogenic signaling pathways. Additionally, its improvement in the gut microbiota profile has been suggested to induce an anti-cancer metabolic profile. Tea compounds increase bacteria that promote the synthesis and metabolism of the beneficial SCFAs and phenolic acids while decreasing bacteria that produce detrimental metabolites including ammonia, polyamines, H_2_S, and secondary BAs (Figure 3). Although the microbial modulation showed beneficial changes, further metabolomics and confirmation studies are needed to validate the results to support further clinical investigation in the future.

## Figures and Tables

**Table 1:** The anti-cancer effects of tea compounds and its regulatory effects on gut microbiota and metabolites. ↓: decrease. ↑: increase. NA: No data available. ACF: aberrant crypt foci. PD-L1: programmed death-ligand 1.

Tea Compounds	Cancers	Gut Microbiota	Effects on Cancer	Citations
Epigallocatechin gallate (EGCG)	Colorectal	*↓ Bacteroides*, *Anaerotruncus*, and *Faecalilbacterium* *↑ Clostridiaceae*, *Fusobacterium*, *Ruminococcus*, *Ochrobactrum*, *Veillonella*, *Desulfococcus*, *Enterobacteriaceae*, and *Sulfurimonas*	↓ Number of ACF	[30]
	Lung	*↓ Deltaproteobacteria* and *Epsilonproteobacteria* *↑ Clostridia*	↓ Cancer nodules growth	[31]
Caffeic acid (CaA)	Liver	*↓ Rumincoccaceae UCG-004* *↑ Lachnospiraceae incertae sedis* and *Prevotella 9*	↓ Liver injury markers ↓ Histopathological changes	[32]
Theabrownin (TB)	Colorectal	*↓ Bacteroidceae* and *Bacteroides* *↑ Prevotellaceae* and *Alloprevotella*	↓ Total tumor count	[33]
Quercetin (QUE)	Colorectal	*↓ Enterobacteriaceae* and *coliform bacteria* *↑ Bifidobacterium* and *Lactobacillus*	↓ Number of ACF and adenoma ↓ Intestinal bleeding	[34]
	Liver	*↑ Firmicutes*, *Actinobacteria*, *VerrucomicrobiotaI*, *Dubosiella*, and *Akkermansia*	↓ Necrosis, fibrosis, and PD-L1 expression	[35]
Quercetin (QUE) derived 3,4-dyhydroxyphenylacetic acid (3,4HPAA)	Colorectal	NA	↓ Malignant transformation ↓ Mitochondrial dysfunction in colon	[36]
Kaempferol	Colorectal	*↓ Firmicutes*, *Eubacterium*, *Anaerophaga*, *Mucispirillum*, *Oscillospira*, *Pseudobutyrivibrio*, *Rikenella*, *Anaerostipes*, *Desulfovibrio*, *Helicobacter*, *Lachnospira*, *Roseburia* and *Ruminococcus* *↓* BA-producing bacteria including *Clostridium lavalense*, *Eubacterium desmolans*, *Eubacterium rectale*, *Intestinimonas butyriciporducens*, *Lachnoclostridium hylemonae* and *Pseudoflavonifractor bacteroides capillosus* *↑ Bacteroidetes*, *Actinobacteria and Verrucomicrobia*, *Akkermansia*, *Alloprevotella*, *Bacteroides*, *Barnesiella*, *Gloebacter*, *Odoribacter*, *Parabacteroides*, *Akkermansia*, *Bacillus*, *Barnesiella*, *Bifidobacterium*, and *Coprococcus*	↓ Tumor burden ↑ Intestinal barrier	[37]
	Lung	*↑ Lachnospiraceae*, *Bacteroidaceae*, *Lactobacillus* and *Bacteroides*	↓ Tumor weight and size	[38]
Spermidine (Spd)	Colorectal	*↓ Proteobacteria* and *Deferribacteres* *↑ Prevotella*	↓ Tumor burden	[39]
